# Publisher Correction: Stable inheritance of H3.3-containing nucleosomes during mitotic cell divisions

**DOI:** 10.1038/s41467-022-31303-6

**Published:** 2022-06-20

**Authors:** Xiaowei Xu, Shoufu Duan, Xu Hua, Zhiming Li, Richard He, Zhiguo Zhang

**Affiliations:** 1 Institute for Cancer Genetics, Columbia University Irving Medical Center, New York, NY USA; 2 Herbert Irving Comprehensive Cancer Center, Columbia University Irving Medical Center, New York, NY USA; 3 Department of Pediatrics, Columbia University Irving Medical Center, New York, NY USA; 4 Department of Genetics and Development, Columbia University Irving Medical Center, New York, NY USA

**Keywords:** Replisome, Epigenetics

Correction to: *Nature Communications* 10.1038/s41467-022-30298-4, published online 06 May 2022.

In this article the author name Zhiguo Zhang was incorrectly written as Zhiguo Zhaang. The original article has been corrected.

