# Supplementary material for: Stable inheritance of H3.3-containing nucleosomes during mitotic cell divisions
Source: Nat Commun. 2022 May 6;13:2514. doi: 10.1038/s41467-022-30298-4 (PMC9076889; doi:10.1038/s41467-022-30298-4)
Supplement: Supplementary file 1 — Supplementary information [file 41467_2022_30298_MOESM1_ESM.pdf]

**Supplementary Information**

# Stable inheritance of H3.3-containing nucleosomes during mitotic cell divisions

Xiaowei Xu[1, 2, 3,4, #], Shoufu Duan[1, 2,3,4, #], Xu Hua[1, 2,3,4], Zhiming Li[1, 2,3,4], Richard He[1, 2,3,4], and Zhiguo Zhang[1,2,3,4*]

[1] Institute for Cancer Genetics, Columbia University Irving Medical Center, New York, NY 10032, USA

[2] Herbert Irving Comprehensive Cancer Center, Columbia University Irving Medical Center, New York, NY 10032, USA

[3] Department of Pediatrics, Columbia University Irving Medical Center, New York, NY 10032, USA

[4] Department of Genetics and Development, Columbia University Irving Medical Center, New York, NY 10032, USA

# These authors contributed equally

[*]Lead contact and corresponding author

This file includes:

Supplementary Figures 1-9

Supplementary Table 1-3

## Supplementary Figures

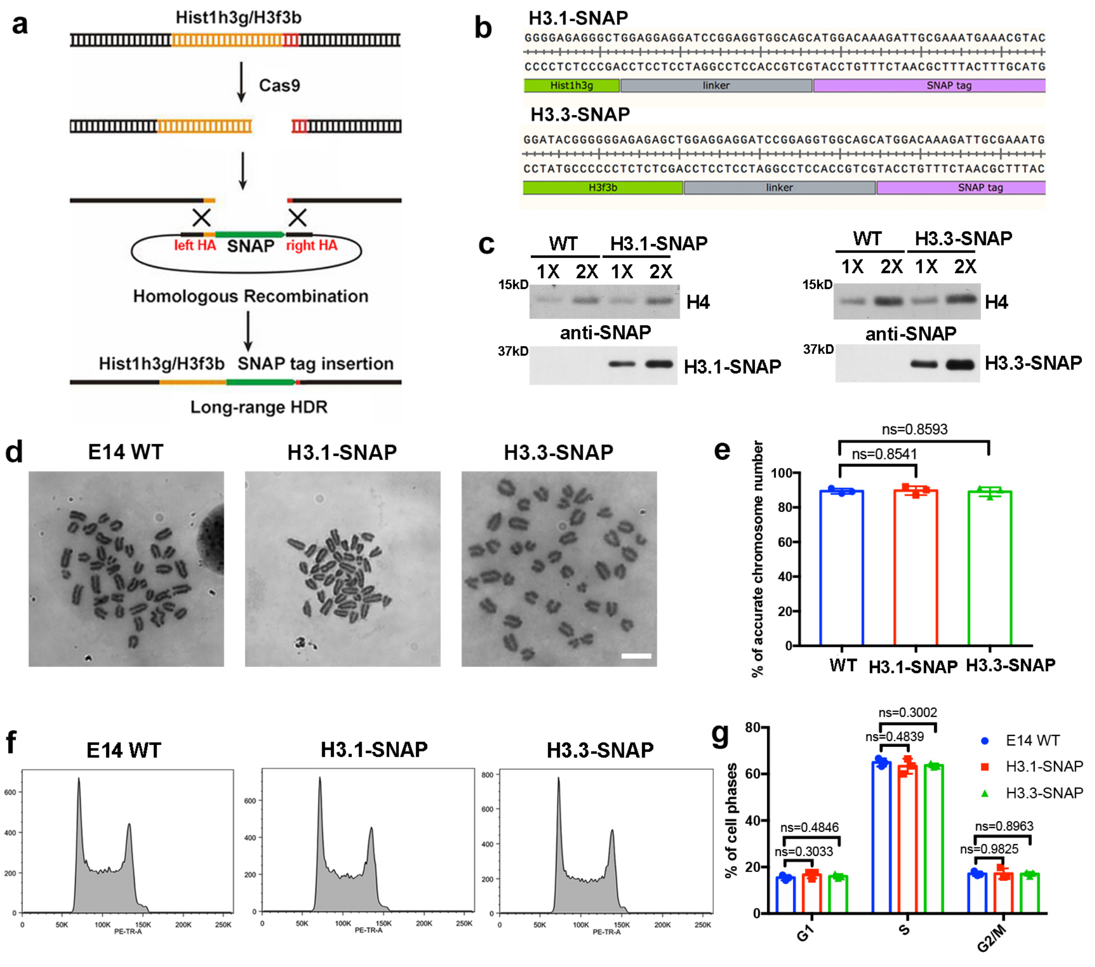


**Supplementary Figure 1: Generation and isolation of H3.1-SNAP and H3.3-SNAP mouse ES cell lines. Related to Figure 1.**

(**a**) Schematic overview of the integration of the SNAP tag sequence immediately after the stop codon of the *Hist1h3g* and *H3f3b* genes, which encode H3.1 and H3.3, respectively.

(**b**) Sanger sequence of the SNAP tag insertion site at the *Hist1h3g* or *H3f3b* gene in mES cells.

(**c**) WB analysis of SNAP expression in H3.1-SNAP and H3.3-SNAP tagged mES cells. Histones were isolated using acid extraction. Wild type ES cells without any tag served as controls. H4 was used as loading control. Representative result from three independent replicates was shown.

(**d**) Karyotype analysis of H3.1-SNAP and H3.3-SNAP cells. Representative images of chromosome spread from WT, H3.1-SNAP and H3.3-SNAP cells. Scale bar, 50 µm.

(**e**) Karyotype analysis of H3.1-SNAP and H3.3-SNAP cells. Quantification of the cells with accurate chromosome number in each cell line.

(**f**) Cell cycle analysis in WT, H3.1-SNAP and H3.3-SNAP cell lines. Cells were collected for flow cytometry analysis of DNA content.

(**g**) The percentages of cells at G1, S and G2/M phase of cell cycle in WT, H3.1-SNAP and H3.3-SNAP cells analyzed in (**f**).

(**e, g**) Data are presented as means ± SD from three independent experiments. Statistical analysis was performed by two-tailed unpaired Student *t* test with *P* values marked on the graphs (ns, no significant difference).

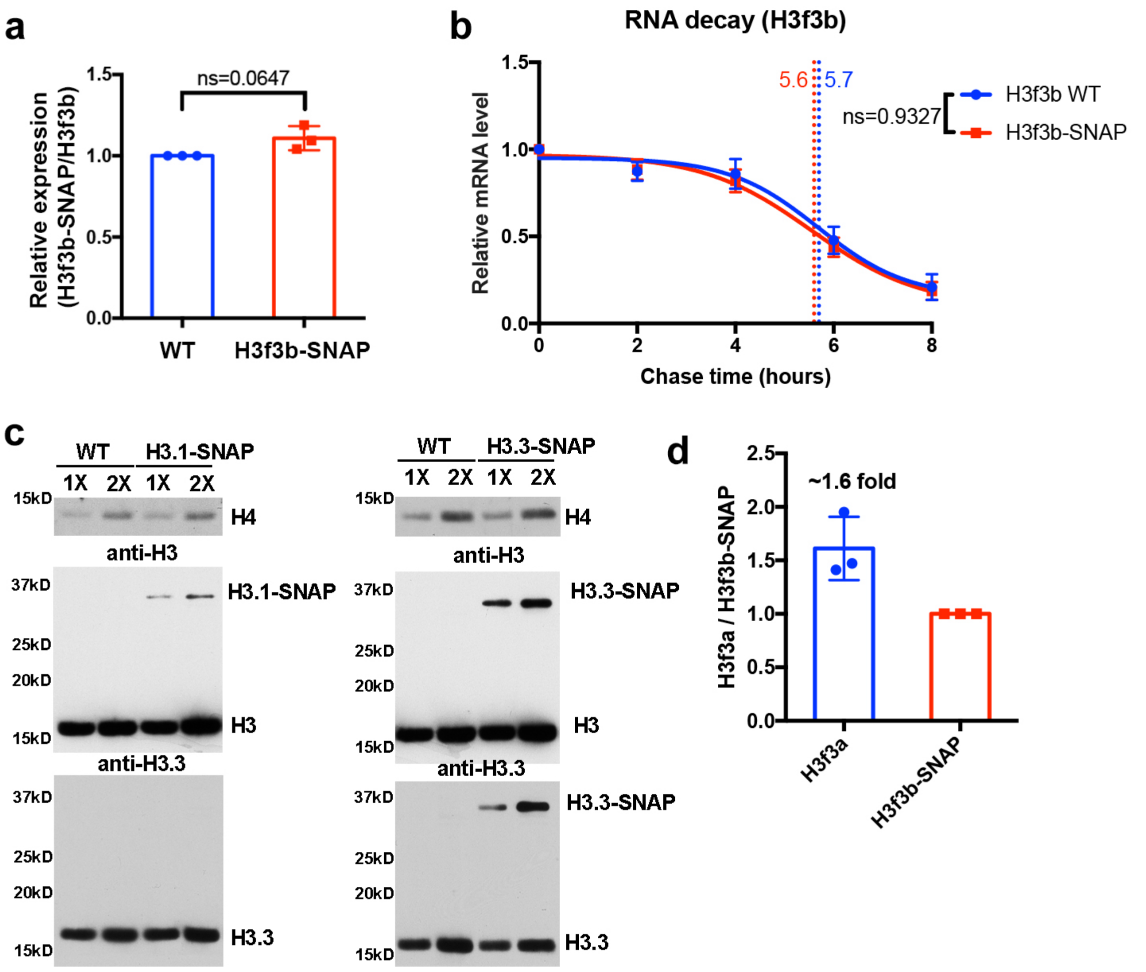


**Supplementary Figure 2: The SNAP tag fusion has no apparent effects on the stability of H3.3 mRNA and protein. Related to Figure 1.**

(**a**) Relative mRNA levels of H3f3b in WT and H3f3b-SNAP cells after normalization to GAPDH. Data are presented as means ± SD from three independent experiments. Statistical analysis was performed by two-tailed unpaired Student *t* test, and *P* values are marked on the graphs (ns, no significant difference).

(**b**) The half-life of H3f3b mRNA is similar to that of H3.3-SNAP. Relative levels of nascent H3f3b mRNA were normalized to GAPDH at each time point. H3f3b mRNA half-life was determined by a fitting of an exponential decay model and labeled in red (H3f3b-SNAP) or blue (WT H3f3b). Data are presented as means ± SD from three independent experiments. Two-tailed unpaired Student *t* test were performed with the *P* values marked on the graphs (ns, no significant difference).

(**c**) WB analysis of H3 and H3.3 in H3.1-SNAP and H3.3-SNAP tagged mES cells.

Histones were isolated by acid extraction. Wild type ES cells without any tag served as controls. H4 was used as loading control. Please note that antibodies against H3.3 recognized H3.3-SNAP, but not H3.1-SNAP. Representative result was shown from three independent replicates.

(**d**) Image J quantification of fold change between untagged H3f3a (H3.3) and SNAP tagged H3f3b based on the anti-H3.3 signals. Data are presented as means ± SD from three independent experiments.

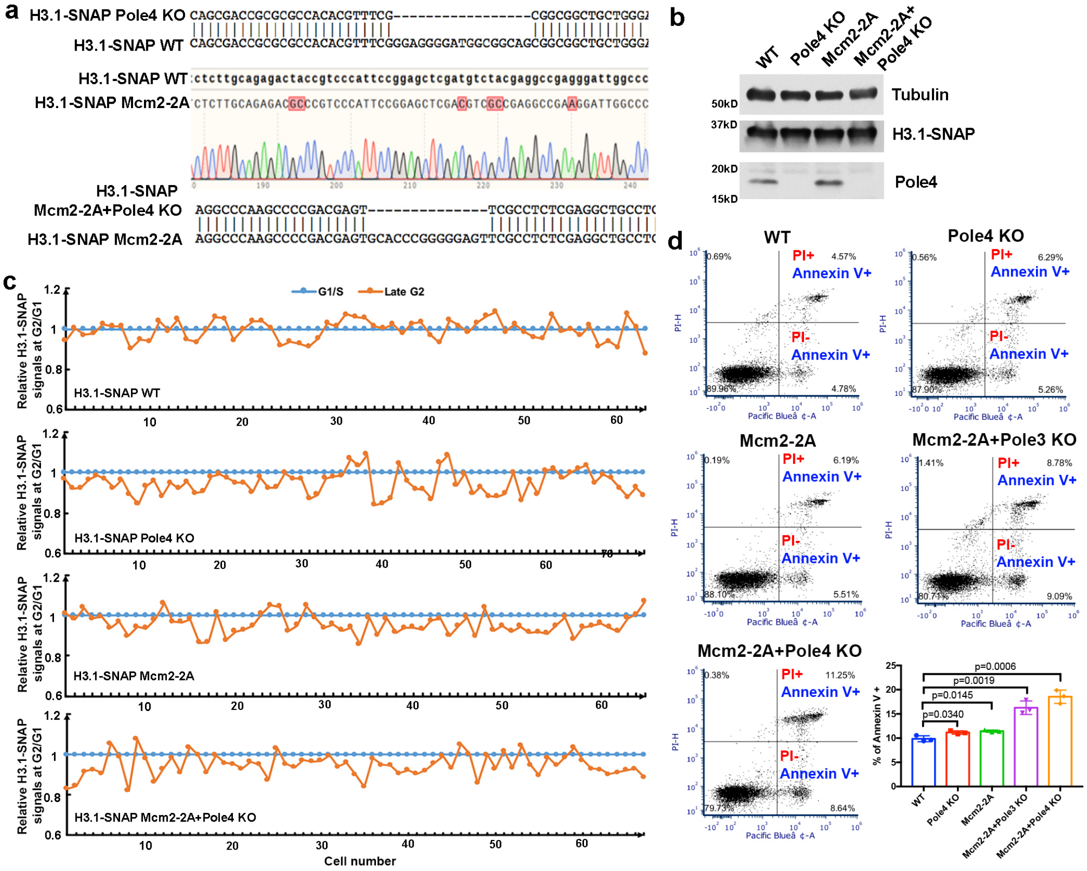

**Supplementary Figure 3: Mcm2 and Pole4 are required for stable recycling of parental H3.1. Related to Figure 2.**

(**a**) Sanger sequencing results of Pole4 KO or Mcm2-2A mutant with nucleotides mutated in Mcm2-2A highlighted in red.

(**b**) Western blot analysis in wild type and various mutant lines to confirm Pole4 KO with H3.1-SNAP (WT, Pole4 KO, Mcm2-2A and Mcm2-2A + Pole4 KO). Representative results from two independent replicates were shown

(**c**) Effects of Pole4 KO, Mcm2-2A or Mcm2-2A + Pole4 KO mutations on the parental H3.1-SNAP signals following DNA replication. Integrated TMR signals in G1/S and G2 of each cell were quantified and normalized to G1/S. X-axis represents number of cell counted, with each dot representing one cell.

(**d**) Mcm2-2A Pole4 KO and Mcm2-2A Pole3 KO double mutant cells show increased apoptosis. Cells were stained and analyzed by flow cytometry. PI+ Annexin V+ group are necrotic cells. PI- Annexin V+ group are apoptotic cells. Lower right: quantification of

Annexin V positive cells (PI+ Annexin V+ plus PI- Annexin V+) ratio in WT, Pole4 KO, Mcm2-2A, Mcm2-2A + Pole3 KO and Mcm2-2A + Pole4 KO cell lines. Data are presented as means ± SD from three independent experiments. Statistical analysis was performed by two-tailed unpaired Student *t* test with *P* values indicated on the graphs (ns, no significant difference).

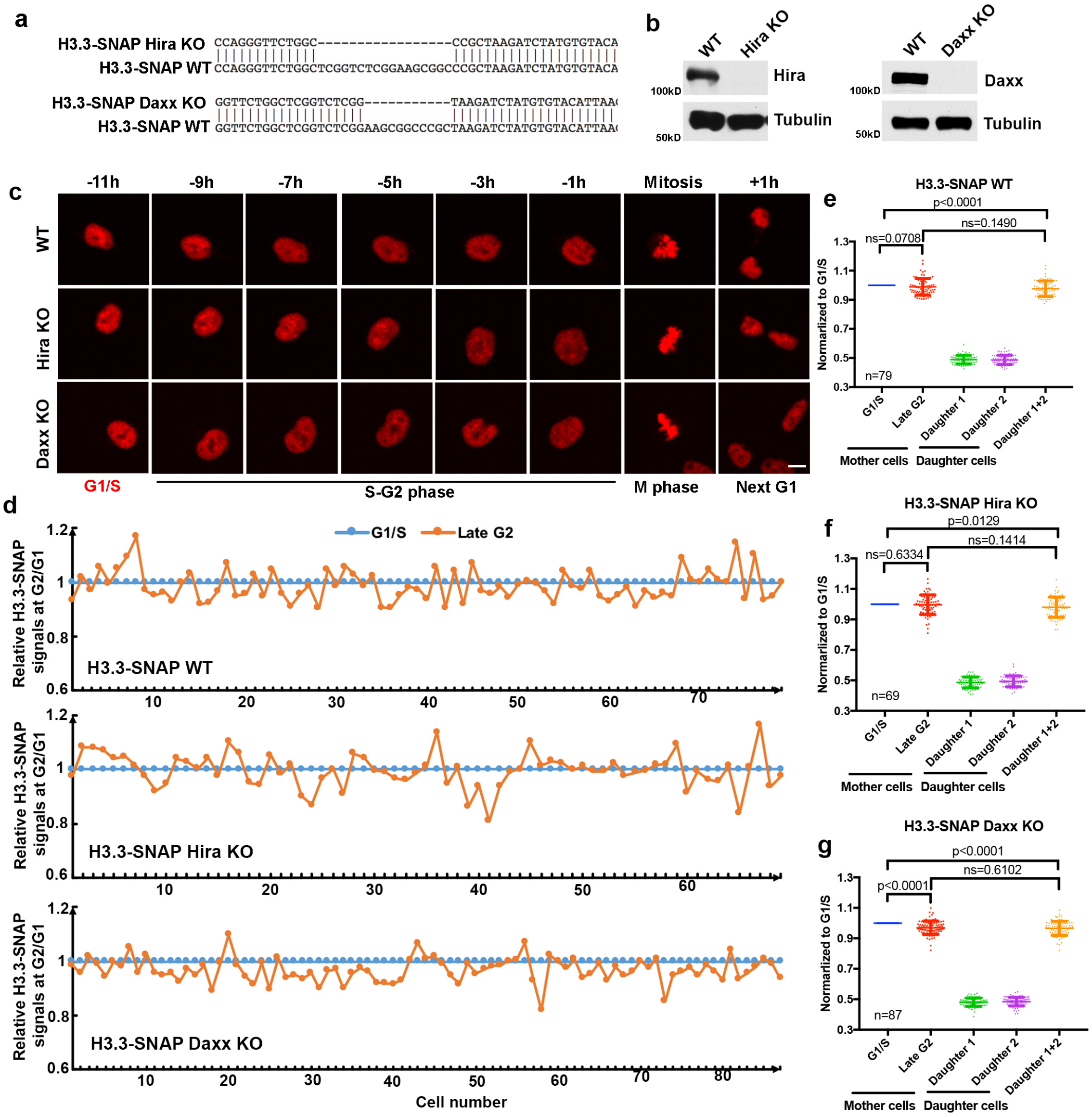


**Supplementary Figure 4: Hira is dispensable for parental H3.3 recycling following DNA replication. Related to Figure 3.**

(**a**) Sanger sequencing analysis of DNA at regions targeted by sgRNA for deletion of Hira or Daxx.

(**b**) Western blot analysis of Hira and Daxx in wild type, Hira KO and Daxx KO cells expressing H3.3-SNAP. Representative results two independent experiments were shown.

(**c**) Representative live cell imaging of TMR labeled parental histones H3.3 at the indicated time points in WT (n=79), Hira KO (n=69) and Daxx KO (n=87) mES cell lines. Scale bar, 10 µm. n, number of cells from two independent experiments.

(**d**) Hira KO did not affect parental H3.3 recycling following DNA replication. Integrated TMR signals at G2 were normalized to G1/S of each individual cell and presented as relative TMR signals at G2/G1 for each cell.

(**e-g**) The average of H3.3-SNAP signals in G1/S, G2 and next G1 of WT, Hira KO and Daxx KO mES cells. Data are presented as means ± SD. Statistical analysis was performed by two-tailed unpaired Student *t* test with *P* values shown on the graphs (ns, no significant difference). n, number of cells from two independent experiments.

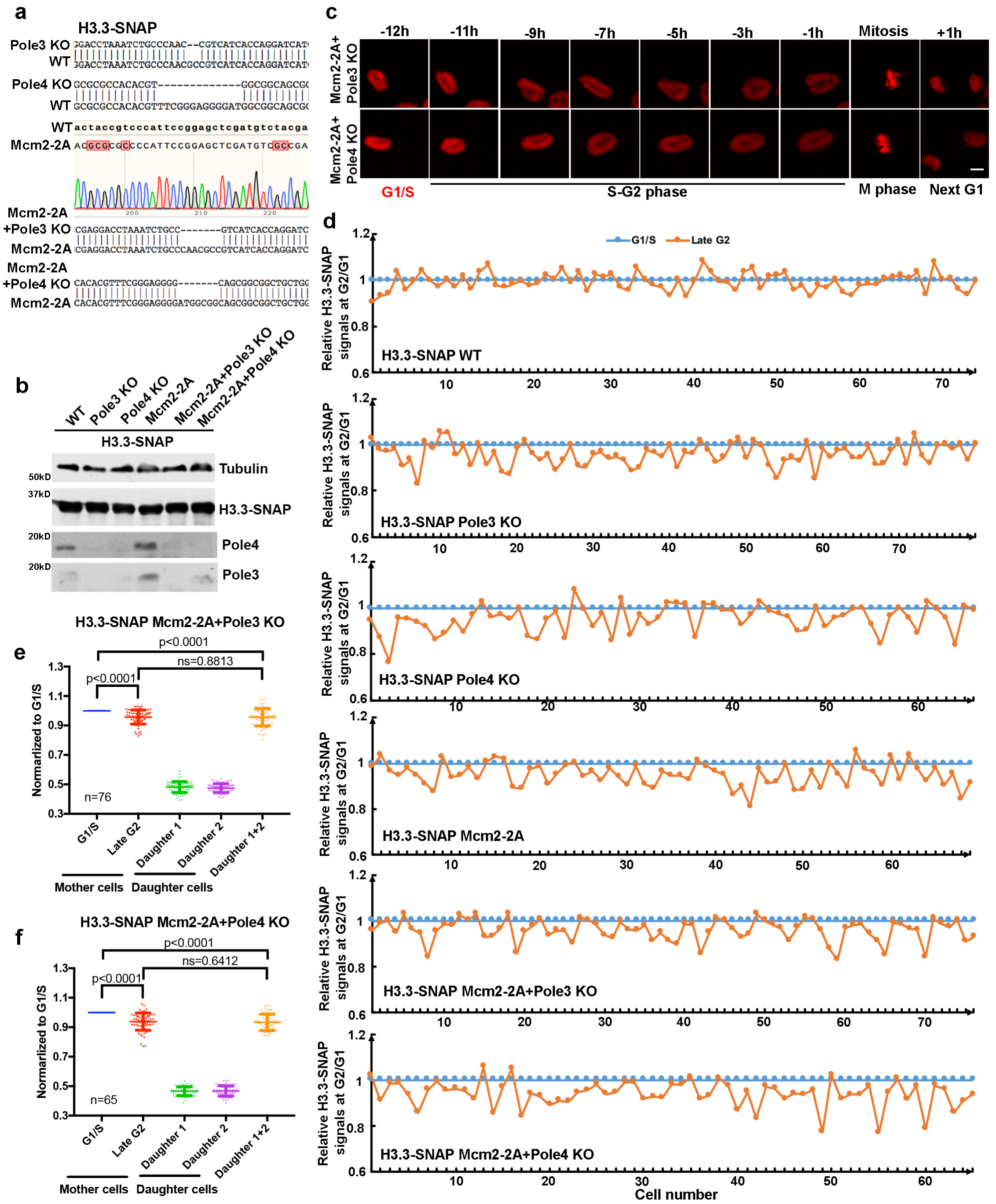


**Supplementary Figure 5: Mcm2, Pole3 and Pole4 are required for recycling of parental H3.3. Related to Figure 3.**

(**a**) Sanger sequencing confirming Pole3 KO, Pole4 KO or Mcm2-2A in H3.3-SNAP lines with nucleotides mutated in Mcm2-2A highlighted in red.

(**b**) Western blot analysis of Pole3 KO and Pole4 KO in indicated cell lines. Representative results from two independent replicates were shown.

(**c**) Representative live cell images of TMR labeled parental H3.3 at the indicated time points in H3.3-SNAP Mcm2-2A + Pole3 KO (n=76) and Mcm2-2A + Pole4 KO (n=65) mES cell lines. n, number of cells from two independent experiments. Scale bar, 10 μm.

(**d**) Effects of Pole3 KO, Pole4 KO, Mcm2-2A, Mcm2-2A + Pole3 KO, Mcm2-2A +Pole4 on the relative integrated TMR signals at G2 compared to G1/S at each individual cells.

(**e, f**) The average of parental H3.3-SNAP signals at G1/S, G2, and two daughter cells in Mcm2-2A + Pole3 KO mES and Mcm2-2A + Pole4 KO mES cells. Data are presented as means ± SD with the number of cells (n) from two independent experiments. Statistical analysis was performed using two-tailed unpaired Student *t* test. The *P* values are marked on the graphs (ns, no significant difference).

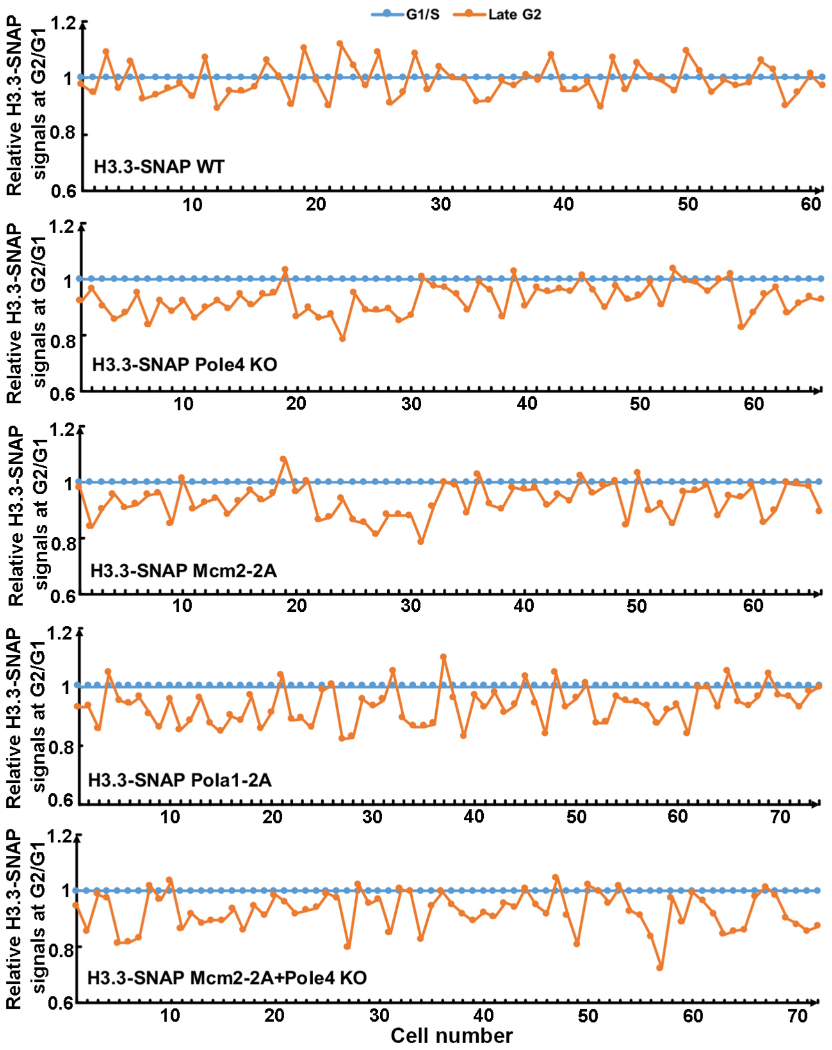


**Supplementary Figure 6: Mcm2, Pola1, Pole3 and Pole4 participate in the recycling of parental H3.3. Related to Figure 4.**

Effects of Pole4 KO, Mcm2-2A, Pola1-2A and Mcm2-2A + Pole4 KO double mutations on the parental H3.3-SNAP signals following DNA replication. Integrated H3.3-SNAP signals detected by 647-SiR (far red) in each individual cells at G2 were quantified and normalized against those at G1/S. X-axis represents number of cell counted, with each dot representing one cell. The G1/S transitions were defined by mAG-Geminin as described in Fig. 4.

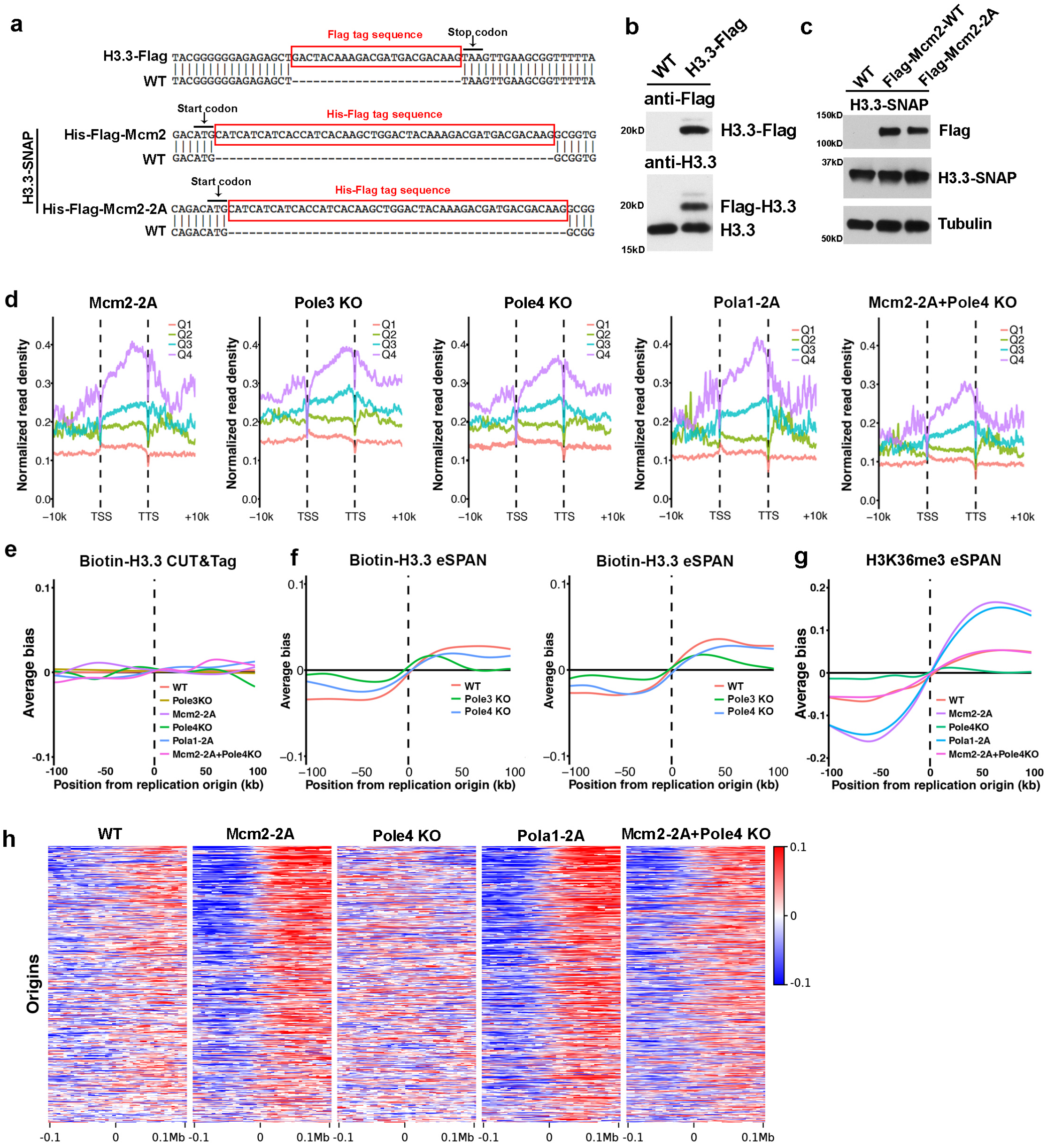


**Supplementary Figure 7: Effects of Pole3 and Pole4 deletion alone and in combination with Mcm2-2A on the distribution of parental H3.3-SNAP and H3K36me3 at replicating DNA strands. Related to Figure 5.**

(**a**) Sanger sequencing confirming H3.3 tagged with the Flag epitope, Mcm2 WT and Mcm2-2A mutant tagged with His-Flag epitope, with the Flag tag or His-Flag tag sequences highlighted in red boxes.

(**b**) Western blot analysis confirming H3.3-Flag expression. Representative results from two independent replicates were shown.

(**c**) Western blot analysis confirming the expression of His-Flag-Mcm2 and His-Flag-Mcm2-2A. Representative result from two independent replicates were shown

(**d**) The distribution of parental H3.3-SNAP CUT&Tag signals at TSS and TTS of genes at four different groups (Q1-Q4) in Mcm2-2A, Pole3 KO, Pole4 KO, Pola1-2A and Mcm2-2A+Pole4 KO mES cells. Genes were separated four quartiles according to their expression, with Q1 representing the lowest 25% transcribed genes and Q4 the top 25% highly transcribed genes. One representative experiment is shown.

(**e**) H3.3-SNAP CUT&Tag did not show any bias. The average bias of H3.3-SNAP-biotin CUT&Tag signals around replication origins (n=1,548) is calculated in WT, Pole3 KO, Pole4 KO, Mcm2-2A, Pola1-2A and Mcm2-2A+Pole4 KO mES cells using the formula (W−C)/(W+C). Results from one representative were shown. W: sequencing read of Watson strand, C: Sequencing read of Crick strand.

(**f**) The average bias of parental H3.3-SNAP eSPAN signals at replication origins (n=1,548) in WT, Pole3 KO and Pole4 KO mES cells. Two independent replicates were shown.

(**g**) Effects of Mcm2-2A, Pola1-2A, Pole4 KO and Mcm2-2A+Pole4 KO double mutant cells on the distribution of H3K36me3 at replicating DNA chromatin. The average bias of H3K36me3 eSPAN at 1,548 replication origins in WT, Mcm2-2A, Pole4 KO, Pola1-2A and Mcm2-2A+Pole4 KO mES cells. One representative experiment is shown.

(**h**) Heatmaps of parental H3K36me3 eSPAN bias at each of the 1,548 initiation zones in WT, Mcm2-2A, Pole4 KO, Pola1-2A and Mcm2-2A+Pole4 KO mES cells, ranked from the most efficient (top) to the least efficient (bottom) ones based on OK-seq.

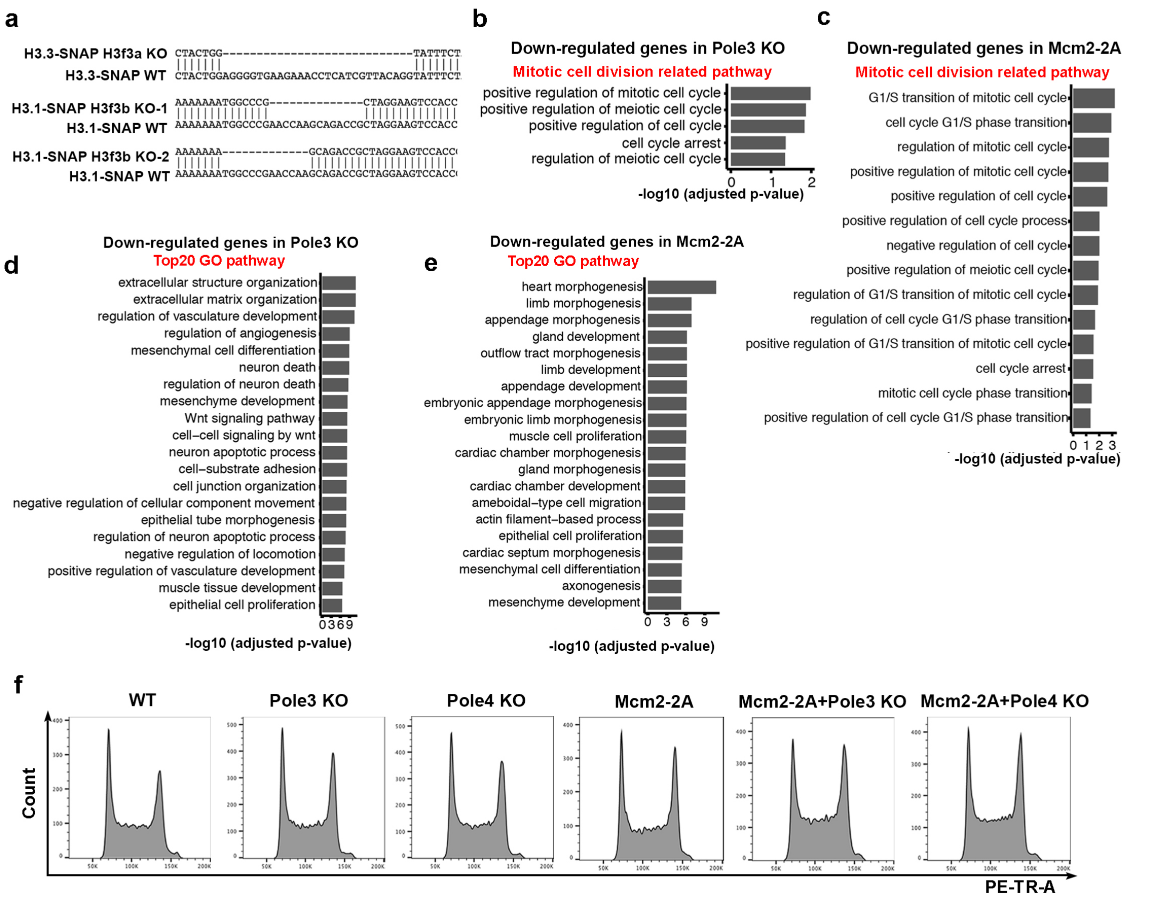


**Supplementary Figure 8: Gene ontology analysis of down-regulated genes in Mcm2-2A and Pole3 KO cells. Related to Figure 7.**

(**a**) Sanger sequencing confirming deletion of H3f3a or H3f3b gene compared to wild type sequencing.

(**b, c**) GO analysis of down-regulated genes in Pole3 KO cells and Mcm2-2A mutant cells based on RNA-seq analysis with cell cycle related pathways shown.

(**d, e**) GO analysis of down-regulated genes in Pole3 KO cells and Mcm2-3A mutant cels based on RNA-seq analysis, with top 20 pathways shown. Please note that All cell lines used in the RNA-seq analysis contain H3.1-SNAP tag.

(**f**) Cell cycle analysis in WT, Pole3 KO, Pole4 KO, Mcm2-2A, Mcm2-2A + Pole3 KO and Mcm2-2A + Pole4 KO cell lines. Cells were collected for flow cytometry analysis of DNA content.

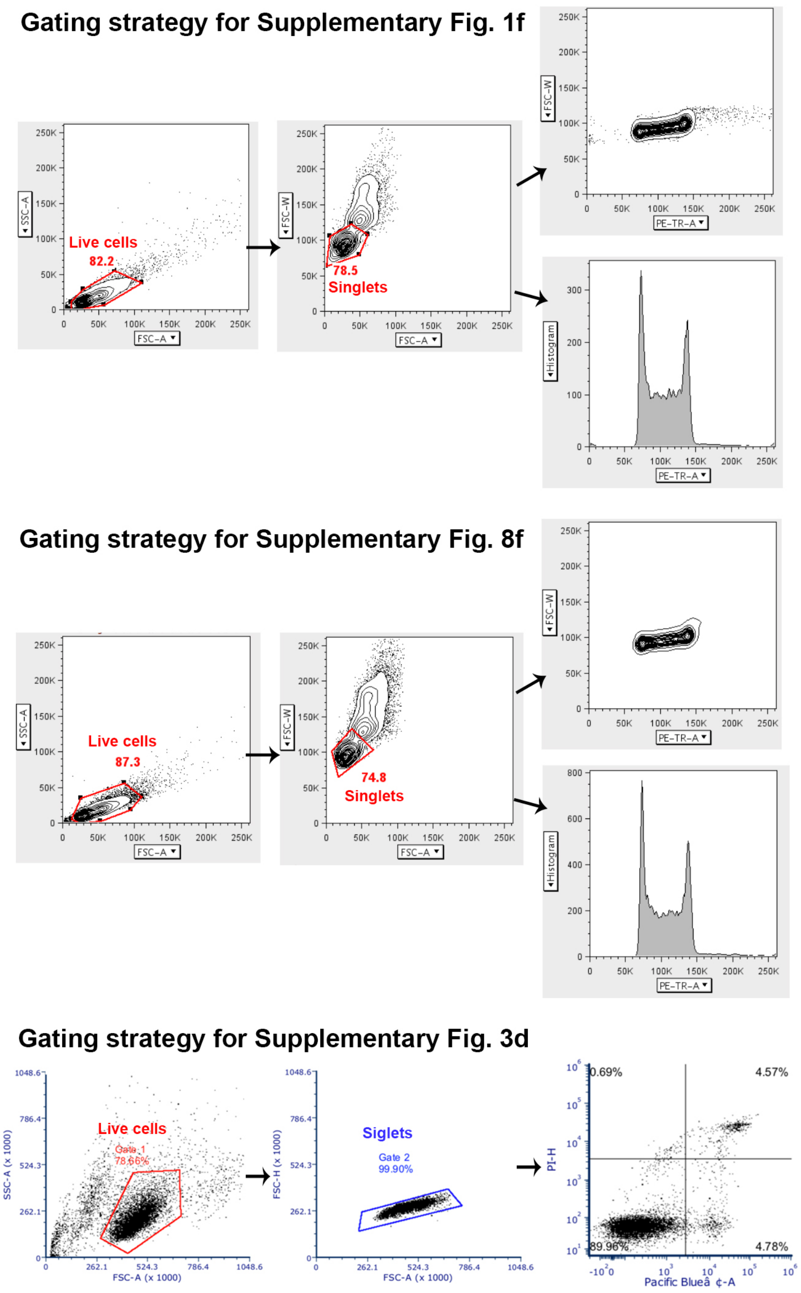


**Supplementary Figure 9: Gating strategies of FACS experiments.**

## Supplementary Tables

### Supplementary Table 1. Oligos used in this study.

| Oligo name | Forward | Reverse |
|---|---|---|
| H3.1 (Hist1h3g) sgRNA | TCCGCGGGGAGAGGGCTTA | TTAAGCCCTCTCCCCGCGGA |
| H3.3 (H3f3b) sgRNA | GAGAGAGCTTAAGTTGAAG | CTTCAACTTAAGCTCTCTC |
| pBlueScript II | CTAGTTCTAGAGCGGCCGCCACCGC | TGGATCCCCCGGGCTGCAGGAATTC |
| SNAP tag (H3.1) | GGAGGAGGATCCGGAGGTGGCAGCA TGGACAAAGATTGCGAAAT | TTGTGTGGATTAACAGAAACCCTTA TCCCAGACCCGGTTTACCCA |
| H3.1 left HA | GAATTCCTGCAGCCCGGGGGATCCAA TATTTTCCAGAGTGGTTTG | GCTGCCACCTCCGGATCCTCC TCCAGCCCTCTCCCCGCGGATGC |
| H3.1 right HA | TAAGGGTTTCTGTTAATCCACACA A | GCGGTGGCGGCCGCTCTAGAACTA GTTTTCAGCATCCAGAGTTCG |
| SNAP tag (H3.3) | GGAGGAGGATCCGGAGGTGGCAGCA TGGACAAAGATTGCGAAAT | ATGCCATAAAAACCGCTTCAACTTA TCCCAGACCCGGTTTACCCA |
| H3.3 left HA | AATTCCTGCAGCCCGGGGGATCCATC CTGTCTGCTTCCCGGTGG | GCTGCCACCTCCGGATCCTCCTCCA GCTCTCTCCCCCCGTATCC |
| H3.3 right HA | TAAGTTGAAGCGGTTTTTATGGCAT | GCGGTGGCGGCCGCTCTAGAACTA GGAACTCAATCTAGGCCTAAG |
| H3.1-Flag donor oligo | TCATGCCCAAGGACATCCAGCTGGCCCGTCGCATCCGCGGGGAGAGGGCTGA CTACAAAGACGATGACGACAAGTAAGGGTTTCTGTTAATCCACACAACCAC TTTAAAGGCTCTTCTTAGAGC | |
| H3.3-Flag donor oligo | CAAAGACATCCAGTTGGCTCGCCGGATACGGGGGGAGAGAGCTGACTACAAA GACGATGACGACAAGTAAGTTGAAGCGGTTTTTATGGCATTTTGTAGTAAA TTCTGTAAAATACTTTGGTTT | |
| Mosaic end adaptor A | TCGTCGGCAGCGTCAGATGTGTATAAGAGACAG | |
| Mosaic end adaptor B | /phos/CTGTCTCTTATACACATCTCCGAGCCCACGAGACinvT | |
| Mosaic end block | /phos/CTGTCTCTTATACAddC | |
| Pole3 KO sgRNA | ATCCTGGTGATGACGGCGTT | AACGCCGTCATCACCAGGAT |
| Pole4 KO sgRNA | CACGTTTCGGGAGGGGATGG | CCATCCCCTCCCGAAACGTG |
| Mcm2-2A sgRNA | CATCGAGCTCCGGAATGGGA | TCCCATTCCGGAGCTCGATG |
| Mcm2-2A donor oligo | CCTAATTCTTAAGTGGGTTGTTTGGGGATTCATTGTCCACTGTTGGTCTCTTGC AGAGACGCGCGCCCCATTCCGGAGCTCGATGTCGCCGAGGCCGAGGGATTGG CCCTGGATGATGAAGATGTGGAGGAGCTGACAGCCAGTCAGAG | |

| | | |
|---|---|---|
| Mcm2-Flag sgRNA | CGGTGGAGCTAGCGCAGACA | TGTCTGCGCTAGCTCCACCG |
| Mcm2-Flag donor oligo | GCGAAAACCGGTGGCAGCTGGTGCGGTGGAGCTAGCGCAGACATGCATCATCATCACCATCACAAGCTGGACTACAAAGACGATGACGACAAGGCGGTGAGGAATGGGCTGATCCCGGGCGGGCGGTATTCAGCGATC | |
| Hira KO sgRNA | TTCATTGTTCGGCCGCTGCC | GGCAGCGGCCGAACAATGAA |
| Daxx KO sgRNA | TTCTGGCTCGGTCTCGGAAG | CTTCCGAGACCGAGCCAGAA |
| H3f3a KO sgRNA | TAGAAATACCTGTAACGATG | CATCGTTACAGGTATTTCTA |
| H3f3b KO sgRNA | TCCTAGCGGTCTGCTTGGTT | AACCAAGCAGACCGCTAGGA |
| H3f3b RT | CCAAGGCGGCTCGGAAAAGC | GGTAACGACGGATCTCTCTCAGA |
| GAPDH RT | CTGACGTGCCGCCTGGAGAAAC | CCCGGCATCGAAGGTGGAAGAGT |

**Supplementary Table 2. GO annotations of cell cycle related pathways for down-regulated genes in Mcm2-2A mutants.**

GO/KEGG enrichment test is a one-sided version of Fisher's exact test. Benjamini-Hochberg adjustments are made for multiple comparisons.

| ID | Description | P value | Gene ID |
|---|---|---|---|
| GO:0000082 | G1/S transition of mitotic cell cycle | 6.32E-04 | Cdkn1b/Hacd1/Pim2/Ube2e2/Cables1/Adamts1/Camk2d/Bcl2/Egfr/Id2 |
| GO:0044843 | cell cycle G1/S phase transition | 1.15E-03 | Cdkn1b/Hacd1/Pim2/Ube2e2/Cables1/Adamts1/Camk2d/Bcl2/Egfr/Id2 |
| GO:0007346 | regulation of mitotic cell cycle | 1.75E-03 | Fgfr1/Gnai1/Cdkn1b/Igf1r/Hacd1/Top2b/Stat5b/Ptch1/Ube2e2/Adamts1/Camk2d/Bcl2/Foxc1/Egfr/Pttg1/Id2/Fgf8/Tnf |
| GO:0045931 | positive regulation of mitotic cell cycle | 1.97E-03 | Fgfr1/Igf1r/Stat5b/Ube2e2/Adamts1/Camk2d/Egfr/Fgf8/Tnf |
| GO:0045787 | positive regulation of cell cycle | 2.39E-03 | Fgfr1/Lfng/Cdkn1b/Igf1r/Stat5b/Ube2e2/Adamts1/Camk2d/Egfr/Id2/Fgf8/Wnt4/Tnf/Tgfb2 |
| GO:0090068 | positive regulation of cell cycle process | 9.47E-03 | Fgfr1/Igf1r/Ube2e2/Adamts1/Camk2d/Egfr/Id2/Fgf8/Wnt4/Tnf |
| GO:0045786 | negative regulation of cell cycle | 9.57E-03 | Cdkn1b/Ilk/Pea15a/Rhob/Top2b/Cgrrf1/Mapk12/Bcl2/Foxc1/Egfr/Pttg1/Id2/Thbs1/Tnf/Tgfb2 |
| GO:0051446 | positive regulation of meiotic cell cycle | 1.11E-02 | Lfng/Igf1r/Wnt4 |
| GO:2000045 | regulation of G1/S transition of mitotic cell cycle | 1.24E-02 | Hacd1/Ube2e2/Adamts1/Bcl2/Egfr/Id2 |
| GO:1902806 | regulation of cell cycle G1/S phase transition | 2.08E-02 | Hacd1/Ube2e2/Adamts1/Bcl2/Egfr/Id2 |
| GO:1900087 | positive regulation of G1/S transition of mitotic cell cycle | 2.72E-02 | Ube2e2/Adamts1/Egfr |
| GO:0007050 | cell cycle arrest | 2.87E-02 | Cdkn1b/Ilk/Cgrrf1/Id2/Thbs1/Tgfb2 |
| GO:0044772 | mitotic cell cycle phase transition | 3.81E-02 | Cdkn1b/Hacd1/Pim2/Ube2e2/Cables1/Adamts1/Camk2d/Bcl2/Cdk14/Egfr/Id2 |
| GO:1902808 | positive regulation of cell cycle G1/S phase transition | 4.61E-02 | Ube2e2/Adamts1/Egfr |

**Supplementary Table 3. GO annotations of pathways related to cell cycle pathways for down-regulated genes in Pole3 KO mutants.**

GO/KEGG enrichment test is a one-sided version of Fisher's exact test. Benjamini-Hochberg adjustments are made for multiple comparisons.

| ID | Description | P value | Gene ID |
|---|---|---|---|
| GO:0045931 | positive regulation of mitotic cell cycle | 1.00E-02 | Dusp3/Igf1r/Stat5b/Shb/App/Asns/Insr/Ube2e2/Anxa1/Camk2d/Fgf8/Smoc2 |
| GO:0051446 | positive regulation of meiotic cell cycle | 1.36E-02 | Igf1r/Meioc/Insr/Wnt4 |
| GO:0045787 | positive regulation of cell cycle | 1.47E-02 | Dusp3/Cdkn1b/Cited2/Igf1r/Stat5b/Shb/App/Sox4/Meioc/Pim1/Asns/Insr/Ube2e2/Anxa1/Gdpd5/Camk2d/Fgf8/Wnt4/Smoc2/Tgfb2 |
| GO:0007050 | cell cycle arrest | 4.33E-02 | Cdkn1b/Bin1/Sox4/Apbb1/Ddit3/Brinp2/Thbs1/Pmp22/Tgfb2 |
| GO:0051445 | regulation of meiotic cell cycle | 4.48E-02 | Igf1r/Meioc/Insr/Rps6ka2/Wnt4 |
