# Supplementary material for: Stable inheritance of H3.3-containing nucleosomes during mitotic cell divisions
Source: Nat Commun. 2022 May 6;13:2514. doi: 10.1038/s41467-022-30298-4 (PMC9076889; doi:10.1038/s41467-022-30298-4)
Supplement: Supplementary file 3 — Description of additional Supplementary File [file 41467_2022_30298_MOESM3_ESM.pdf]

Descriptions of additional Supplementary files

Supplementary Movie 1: Live cell images of TMR-labeled parental histone H3.1-SNAP in WT mES cells. Scale bar, 10 µm.

Supplementary Movie 2: Live cell images of TMR-labeled parental histone H3.3-SNAP in WT mES cells. Scale bar, 10 µm.

Supplementary Movie 3: Live cell images of TMR-labeled parental histone H3.1-SNAP in WT mES cells. Scale bar, 10 µm.

Supplementary Movie 4: Live cell images of TMR-labeled parental histone H3.1-SNAP in Pole4 KO mES cells. Scale bar, 10 µm.

Supplementary Movie 5: Live cell images of TMR-labeled parental histone H3.1-SNAP in Mcm2-2A mES cells. Scale bar, 10 µm.

Supplementary Movie 6: Live cell images of TMR-labeled parental histone H3.1-SNAP in Mcm2-2A + Pole4 KO mES cells. Scale bar, 10 µm.

Supplementary Movie 7: Live cell images of TMR-labeled parental histone H3.3-SNAP in WT mES cells. Scale bar, 10 µm.

Supplementary Movie 8: Live cell images of TMR-labeled parental histone H3.3-SNAP in Hira KO mES cells. Scale bar, 10 µm.

Supplementary Movie 9: Live cell images of TMR-labeled parental histone H3.3-SNAP in Daxx KO mES cells. Scale bar, 10 µm.

Supplementary Movie 10: Live cell images of TMR-labeled parental histone H3.3-SNAP in WT mES cells. Scale bar, 10 µm.

Supplementary Movie 11: Live cell images of TMR-labeled parental histone H3.3-SNAP in Pole3 KO mES cells. Scale bar, 10 µm.

Supplementary Movie 12: Live cell images of TMR-labeled parental histone H3.3-SNAP in Pole4 KO mES cells. Scale bar, 10 µm.

Supplementary Movie 13: Live cell images of TMR-labeled parental histone H3.3-SNAP in Mcm2-2A mES cells. Scale bar, 10 µm.

Supplementary Movie 14: Live cell images of TMR-labeled parental histone H3.3-SNAP in Mcm2-2A + Pole3 KO mES cells. Scale bar, 10 µm.

Supplementary Movie 15: Live cell images of TMR-labeled parental histone H3.3-SNAP in Mcm2-2A+Pole4 KO mES cells. Scale bar, 10 µm.

Supplementary Movie 16: Live cell images of 647-SiR-labeled parental histone H3.3-SNAP and mAG tagged geminin in WT mES cells. Scale bar, 10 µm.

Supplementary Movie 17: Live cell images of 647-SiR-labeled parental histone H3.3-SNAP and mAG tagged geminin in Pole4 KO mES cells. Scale bar, 10 µm.

Supplementary Movie 18: Live cell images of 647-SiR-labeled parental histone H3.3-SNAP and mAG tagged geminin in Mcm2-2A mES cells. Scale bar, 10 µm.

Supplementary Movie 19: Live cell images of 647-SiR-labeled parental histone H3.3-SNAP and mAG tagged geminin in Mcm2-2A + Pole4 KO mES cells. Scale bar, 10 µm.

Supplementary Movie 20: Live cell images of 647-SiR-labeled parental histone H3.3-SNAP and mAG tagged geminin in in Pola1-2A mES cells. Scale bar, 10 µm.
