# Supplementary material for: Stable inheritance of H3.3-containing nucleosomes during mitotic cell divisions
Source: Nat Commun. 2022 May 6;13:2514. doi: 10.1038/s41467-022-30298-4 (PMC9076889; doi:10.1038/s41467-022-30298-4)
Supplement: Supplementary file 24 — Reporting Summary [file 41467_2022_30298_MOESM24_ESM.pdf]

Corresponding author(s): Zhiguo Zhang

Last updated by author(s): Apr 12, 2022

Reporting Summary

Nature Portfolio wishes to improve the reproducibility of the work that we publish. This form provides structure for consistency and transparency in reporting. For further information on Nature Portfolio policies, see our Editorial Policies and the Editorial Policy Checklist.

Statistics

For all statistical analyses, confirm that the following items are present in the figure legend, table legend, main text, or Methods section.

n/a	Confirmed	
☐	☒	The exact sample size (*n*) for each experimental group/condition, given as a discrete number and unit of measurement
☐	☒	A statement on whether measurements were taken from distinct samples or whether the same sample was measured repeatedly
☐	☒	The statistical test(s) used AND whether they are one- or two-sided<br>*Only common tests should be described solely by name; describe more complex techniques in the Methods section.*
☒	☐	A description of all covariates tested
☒	☐	A description of any assumptions or corrections, such as tests of normality and adjustment for multiple comparisons
☐	☒	A full description of the statistical parameters including central tendency (e.g. means) or other basic estimates (e.g. regression coefficient) AND variation (e.g. standard deviation) or associated estimates of uncertainty (e.g. confidence intervals)
☐	☒	For null hypothesis testing, the test statistic (e.g. *F*, *t*, *r*) with confidence intervals, effect sizes, degrees of freedom and *P* value noted<br>*Give P values as exact values whenever suitable.*
☒	☐	For Bayesian analysis, information on the choice of priors and Markov chain Monte Carlo settings
☒	☐	For hierarchical and complex designs, identification of the appropriate level for tests and full reporting of outcomes
☐	☒	Estimates of effect sizes (e.g. Cohen's *d*, Pearson's *r*), indicating how they were calculated

Our web collection on statistics for biologists contains articles on many of the points above.

Software and code

Policy information about availability of computer code

Data collection	NIS Elements software (Nikon) was used for live cell image capture. BD FACSDiva and Attune NxT softwares were used for FACS data collection.<br>Deep sequencing for CUT&Run, CUT&Tag and RNA-seq were performed by the Columbia University Genome Center.
Data analysis	Live cell images were analyzed in Fiji (version 1.0, Fiji=Image J2) sofeware. Flowjo (version 10.7.1) and FCS express (version 7) software were used for analyzing FACS data sets. All the statistical tests were processed in Prism software (version 7) and R software (version 3.6.3). CUT&Tag and CUT&RUN data were analyzed using Trim Galore (version 0.6.7), Bowtie2 (version 2.2.4), SAMtools (version 1.11), Picard Tools (version 2.23.8), deepTools (version 3.2.1), BEDTools (version 2.29.2), bedGraphToBigWig tool (version 4), and SICER2 (version 1.0.3). RNA-seq data were analyzed using STAR (version 2.7.6a), featureCounts (version 2.0.1), edgeR (version 3.34.0) and clusterProfiler package (version 3.18.0).

For manuscripts utilizing custom algorithms or software that are central to the research but not yet described in published literature, software must be made available to editors and reviewers. We strongly encourage code deposition in a community repository (e.g. GitHub). See the Nature Portfolio guidelines for submitting code & software for further information.

Data

Policy information about availability of data

All manuscripts must include a data availability statement. This statement should provide the following information, where applicable:

- Accession codes, unique identifiers, or web links for publicly available datasets
- A description of any restrictions on data availability
- For clinical datasets or third party data, please ensure that the statement adheres to our policy

Data Availability
The supporting data generated in this study are provided in the Supplementary Information file. Additional details on datasets and protocols that support the findings of this study will be made available by the corresponding author upon reasonable request. The raw and processed sequencing data used in this study are available in the Gene Expression Omnibus (GEO) database under accession code GSE183065 (https://www.ncbi.nlm.nih.gov/geo/query/acc.cgi?acc=GSE183065). Previously published RNA-seq data under the accession code GSE142996 (https://www.ncbi.nlm.nih.gov/geo/query/acc.cgi?acc=GSE142996) were also used in this study. The GTF and FASTA files used for Bioinformatics analysis (mm10, GENCODE release M27; hg19, GENCODE release 28) can be downloaded from GENCODE (https://www.gencodegenes.org). Source data were provided with this paper.

Field-specific reporting

Please select the one below that is the best fit for your research. If you are not sure, read the appropriate sections before making your selection.

☒ Life sciences ☐ Behavioural & social sciences ☐ Ecological, evolutionary & environmental sciences

For a reference copy of the document with all sections, see nature.com/documents/nr-reporting-summary-flat.pdf

Life sciences study design

All studies must disclose on these points even when the disclosure is negative.

Sample size	Sample sizes were chosen based on previous experience and common practice in the field.<br>For live cell imaging quantification, we tracked at least 60 cells from two independent experiment which we thought this number is large enough to provide statistical power for data quantification (Salomé Adam et al, Molecular Cell, 2016).<br>For each CUT&RUN, CUT&Tag and eSPAN experiments, we have two biologically independent replicates, which was described for similar experiments in published articles (Yu, C. et al, Science, 2018; Li, Z. et al, Science Advances, 2020).<br>Experiments involved in flow cytometry contained at least three biological replicates, each experiment with 10000 cells analyzed.<br>All the WB experiments were carried out in two to three biologically independent samples. The exact numbers of replicates of each experiment were described in each figure legend.
Data exclusions	There are no data exclusions in this study.
Replication	For each experiment, we have at least two independent replicats. All replicate experiments were concordant.
Randomization	Randomization was not relevant to this study, all tests were in-vitro. Cells were clearly identified based on genotype. Cells were cultured under identical conditions and unbiasedly allocated to well positions and treatments. Samples were harvested, processed and analyzed in random order when possible. Microscopy image acquisition was performed randomly, and cells were randomly chosen for quantification.
Blinding	Blinding was not relevant to this study, as all tests were in-vitro and no subjective rating of data was performed. Experimental conditions were typically evident from the data and parameters such as location of well positions is fixed. Key observations of live cell image results were quantified by two independent persons. Flow cytometry samples were analyzed by quantitative methods that report population averages.

Behavioural & social sciences study design

All studies must disclose on these points even when the disclosure is negative.

Study description	*Briefly describe the study type including whether data are quantitative, qualitative, or mixed-methods (e.g. qualitative cross-sectional, quantitative experimental, mixed-methods case study).*
Research sample	*State the research sample (e.g. Harvard university undergraduates, villagers in rural India) and provide relevant demographic information (e.g. age, sex) and indicate whether the sample is representative. Provide a rationale for the study sample chosen. For studies involving existing datasets, please describe the dataset and source.*
Sampling strategy	*Describe the sampling procedure (e.g. random, snowball, stratified, convenience). Describe the statistical methods that were used to predetermine sample size OR if no sample-size calculation was performed, describe how sample sizes were chosen and provide a rationale for why these sample sizes are sufficient. For qualitative data, please indicate whether data saturation was considered, and what criteria were used to decide that no further sampling was needed.*

Data collection	*Provide details about the data collection procedure, including the instruments or devices used to record the data (e.g. pen and paper, computer, eye tracker, video or audio equipment) whether anyone was present besides the participant(s) and the researcher, and whether the researcher was blind to experimental condition and/or the study hypothesis during data collection.*
Timing	*Indicate the start and stop dates of data collection. If there is a gap between collection periods, state the dates for each sample cohort.*
Data exclusions	*If no data were excluded from the analyses, state so OR if data were excluded, provide the exact number of exclusions and the rationale behind them, indicating whether exclusion criteria were pre-established.*
Non-participation	*State how many participants dropped out/declined participation and the reason(s) given OR provide response rate OR state that no participants dropped out/declined participation.*
Randomization	*If participants were not allocated into experimental groups, state so OR describe how participants were allocated to groups, and if allocation was not random, describe how covariates were controlled.*

Ecological, evolutionary & environmental sciences study design

All studies must disclose on these points even when the disclosure is negative.

Study description	*Briefly describe the study. For quantitative data include treatment factors and interactions, design structure (e.g. factorial, nested, hierarchical), nature and number of experimental units and replicates.*
Research sample	*Describe the research sample (e.g. a group of tagged Passer domesticus, all Stenocereus thurberi within Organ Pipe Cactus National Monument), and provide a rationale for the sample choice. When relevant, describe the organism taxa, source, sex, age range and any manipulations. State what population the sample is meant to represent when applicable. For studies involving existing datasets, describe the data and its source.*
Sampling strategy	*Note the sampling procedure. Describe the statistical methods that were used to predetermine sample size OR if no sample-size calculation was performed, describe how sample sizes were chosen and provide a rationale for why these sample sizes are sufficient.*
Data collection	*Describe the data collection procedure, including who recorded the data and how.*
Timing and spatial scale	*Indicate the start and stop dates of data collection, noting the frequency and periodicity of sampling and providing a rationale for these choices. If there is a gap between collection periods, state the dates for each sample cohort. Specify the spatial scale from which the data are taken*
Data exclusions	*If no data were excluded from the analyses, state so OR if data were excluded, describe the exclusions and the rationale behind them, indicating whether exclusion criteria were pre-established.*
Reproducibility	*Describe the measures taken to verify the reproducibility of experimental findings. For each experiment, note whether any attempts to repeat the experiment failed OR state that all attempts to repeat the experiment were successful.*
Randomization	*Describe how samples/organisms/participants were allocated into groups. If allocation was not random, describe how covariates were controlled. If this is not relevant to your study, explain why.*
Blinding	*Describe the extent of blinding used during data acquisition and analysis. If blinding was not possible, describe why OR explain why blinding was not relevant to your study.*

Did the study involve field work? ☐ Yes ☐ No

Field work, collection and transport

Field conditions	*Describe the study conditions for field work, providing relevant parameters (e.g. temperature, rainfall).*
Location	*State the location of the sampling or experiment, providing relevant parameters (e.g. latitude and longitude, elevation, water depth).*
Access & import/export	*Describe the efforts you have made to access habitats and to collect and import/export your samples in a responsible manner and in compliance with local, national and international laws, noting any permits that were obtained (give the name of the issuing authority, the date of issue, and any identifying information).*
Disturbance	*Describe any disturbance caused by the study and how it was minimized.*

Reporting for specific materials, systems and methods

We require information from authors about some types of materials, experimental systems and methods used in many studies. Here, indicate whether each material, system or method listed is relevant to your study. If you are not sure if a list item applies to your research, read the appropriate section before selecting a response.

Materials & experimental systems

n/a	Involved in the study
[ ]	[x] Antibodies
[ ]	[x] Eukaryotic cell lines
[x]	[ ] Palaeontology and archaeology
[x]	[ ] Animals and other organisms
[x]	[ ] Human research participants
[x]	[ ] Clinical data
[x]	[ ] Dual use research of concern

Methods

n/a	Involved in the study
[ ]	[x] ChIP-seq
[ ]	[x] Flow cytometry
[x]	[ ] MRI-based neuroimaging

Antibodies

Antibodies used

Antibodies used in this study were as follows:
antigen | clone number | cat. number | vendor | application | dilution
anti-SNAP|polyclonal|P9310S |New England Biolabs |WB|1:1000
anti-Tubulin|NS-1|12G10|DSHB|WB|1:3000
anti-Pole3|polyclonal|A6469|Abclonal|WB|1:500
anti-Pole4|polyclonal|A9882|Abclonal|WB|1:1000
anti-DAXX|25C12|#4533|Cell Signaling|WB|1:1000
anti-Hira|WC119|04-1488|Millipore Sigma|WB|1:1000
anti-H3.3|-|C15210011|Diagenode|WB|1:1000
anti-Flag|M2|F1804|Sigma-Aldrich|WB|1:2000
anti-His|HIS.H8|CLH101AP|Cedarlane|WB|1:1000
anti-Mcm2|polyclonal|ab4461|Abcam|WB|1:2000
anti-Biotin|polyclonal|A150-109A|Bethyl|CUT&Tag, CUT&RUN|1:800
anti-BrdU|3D4|555627|BD Biosciences|IP| 1:2000
anti-H3 (generated by immunizing rabbits with a synthetic peptide)|-|MC1906|Cocalico Biologicals|WB|1:3000
anti-H3K36me3|MABI 0333|61021|Active Motif|CUT&Tag|1:200

Validation

We have provided Western blot validation in manuscript for antibodies: anti-SNAP (P9310S, NEB), anti-Tubulin (12G10, DSHB), anti-Pole3 (A6469, Abclonal), anti-Pole4 (A9882, Abclonal), anti-DAXX (#4533, Cell Signaling), anti-Hira (04-1488, Millipore), anti-H3.3 (C15210011, Diagenode), anti-Flag (F1804, Sigma), anti-His (CLH101AP, Cedarlane) and anti-Mcm2 (ab4461, Abcam), anti-H3 (MC1906, Cocalico Biologicals). The correct size of the protein of interest was assessed by protein marker.
The specificities of anti-Pole3/4 antibodies were also tested using knockout cell lines.
We confirmed anti-Biotin antibody with both WB and immunofluorescence.
The specificity of anti-BrdU antibody used for BrdU-IP were confirmed in previous published papers of our lab (Gan, H. et al, Molecular Cell, 2018; Yu, C. et al, Science, 2018; Zhiming Li, Science Advances, 2020).
For validation by the manufacturer and cited papers please see the following websites:
anti-SNAP: https://www.neb.com/products/p9310-anti-snap-tag-antibody-polyclonal#Product%20Information
anti-Tubulin: https://dshb.biology.uiowa.edu/12G10-anti-alpha-tubulin
anti-Pole3: https://abclonal.com/catalog-antibodies/KOValidatedPOLE3RabbitpAb/A6469
anti-Pole4: https://abclonal.com/catalog-antibodies/POLE4PolyclonalAntibody/A9882
anti-DAXX: https://www.cellsignal.com/products/primary-antibodies/daxx-25c12-rabbit-mab/4533
anti-Hira: https://www.emdmillipore.com/US/en/product/Anti-HIRA-Antibody-clone-WC119,MM_NF-04-1488
anti-H3.3: https://www.diagenode.com/en/p/h3-3-monoclonal-antibody
anti-Flag: https://www.sigmaaldrich.com/US/en/product/sigma/f1804
anti-His: https://www.cedarlanelabs.com/Products/Detail/CLH101AP?lob=AllProducts
anti-Mcm2: https://www.abcam.com/mcm2-antibody-ab4461.html
anti-Biotin: https://www.fortislife.com/products/primary-antibodies/rabbit-anti-biotin-antibody/A150-109A
anti-BrdU: https://www.bdbiosciences.com/en-us/products/reagents/flow-cytometry-reagents/research-reagents/single-color-antibodies-ruo/purified-mouse-anti-brdu.555627
anti-H3K36me3: https://www.activemotif.com/catalog/details/61021

Eukaryotic cell lines

Policy information about cell lines

Cell line source(s)

The mouse E14 ES cell line was kindly provided by Dr. Tom Fazzio (University of Massachusetts Medical School).
HEK293T (CRL-3216, ATCC).

Authentication

Mouse E14 cells were used within passage 20. Karyotyping were performed to confrim chromosome stability. Pluripotency markers (Oct4, Nanog) were detected by RT-PCT to confirm the stem state. HEK293T cells were used within passage 10. All cells showed expected cell morphology and growth behaviour. No further authentication was performed.

Mycoplasma contamination

All cell lines were tested negative for mycoplasma contamination monthly.

Commonly misidentified lines (See ICLAC register)	No cell lines from the ICLAC register were used.

Palaeontology and Archaeology

Specimen provenance	*Provide provenance information for specimens and describe permits that were obtained for the work (including the name of the issuing authority, the date of issue, and any identifying information). Permits should encompass collection and, where applicable, export.*
Specimen deposition	*Indicate where the specimens have been deposited to permit free access by other researchers.*
Dating methods	*If new dates are provided, describe how they were obtained (e.g. collection, storage, sample pretreatment and measurement), where they were obtained (i.e. lab name), the calibration program and the protocol for quality assurance OR state that no new dates are provided.*

☐ Tick this box to confirm that the raw and calibrated dates are available in the paper or in Supplementary Information.

Ethics oversight	*Identify the organization(s) that approved or provided guidance on the study protocol, OR state that no ethical approval or guidance was required and explain why not.*

Note that full information on the approval of the study protocol must also be provided in the manuscript.

Animals and other organisms

Policy information about studies involving animals; ARRIVE guidelines recommended for reporting animal research

Laboratory animals	*For laboratory animals, report species, strain, sex and age OR state that the study did not involve laboratory animals.*
Wild animals	*Provide details on animals observed in or captured in the field; report species, sex and age where possible. Describe how animals were caught and transported and what happened to captive animals after the study (if killed, explain why and describe method; if released, say where and when) OR state that the study did not involve wild animals.*
Field-collected samples	*For laboratory work with field-collected samples, describe all relevant parameters such as housing, maintenance, temperature, photoperiod and end-of-experiment protocol OR state that the study did not involve samples collected from the field.*
Ethics oversight	*Identify the organization(s) that approved or provided guidance on the study protocol, OR state that no ethical approval or guidance was required and explain why not.*

Note that full information on the approval of the study protocol must also be provided in the manuscript.

Human research participants

Policy information about studies involving human research participants

Population characteristics	*Describe the covariate-relevant population characteristics of the human research participants (e.g. age, gender, genotypic information, past and current diagnosis and treatment categories). If you filled out the behavioural & social sciences study design questions and have nothing to add here, write "See above."*
Recruitment	*Describe how participants were recruited. Outline any potential self-selection bias or other biases that may be present and how these are likely to impact results.*
Ethics oversight	*Identify the organization(s) that approved the study protocol.*

Note that full information on the approval of the study protocol must also be provided in the manuscript.

Clinical data

Policy information about clinical studies

All manuscripts should comply with the ICMJEguidelines for publication of clinical research and a completedCONSORT checklist must be included with all submissions.

Clinical trial registration	*Provide the trial registration number from ClinicalTrials.gov or an equivalent agency.*
Study protocol	*Note where the full trial protocol can be accessed OR if not available, explain why.*
Data collection	*Describe the settings and locales of data collection, noting the time periods of recruitment and data collection.*
Outcomes	*Describe how you pre-defined primary and secondary outcome measures and how you assessed these measures.*

Dual use research of concern

Policy information about dual use research of concern

Hazards

Could the accidental, deliberate or reckless misuse of agents or technologies generated in the work, or the application of information presented in the manuscript, pose a threat to:

No	Yes	
☒	☐	Public health
☒	☐	National security
☒	☐	Crops and/or livestock
☒	☐	Ecosystems
☒	☐	Any other significant area

Experiments of concern

Does the work involve any of these experiments of concern:

No	Yes	
☒	☐	Demonstrate how to render a vaccine ineffective
☒	☐	Confer resistance to therapeutically useful antibiotics or antiviral agents
☒	☐	Enhance the virulence of a pathogen or render a nonpathogen virulent
☒	☐	Increase transmissibility of a pathogen
☒	☐	Alter the host range of a pathogen
☒	☐	Enable evasion of diagnostic/detection modalities
☒	☐	Enable the weaponization of a biological agent or toxin
☒	☐	Any other potentially harmful combination of experiments and agents

ChIP-seq

Data deposition

☒ Confirm that both raw and final processed data have been deposited in a public database such as GEO.

☒ Confirm that you have deposited or provided access to graph files (e.g. BED files) for the called peaks.

Data access links
May remain private before publication.

https://www.ncbi.nlm.nih.gov/geo/query/acc.cgi?acc=GSE183065

Files in database submission

GSM5550386 WT_H33_CUT_rep1
GSM5550387 Mcm22A_H33_CUT_rep1
GSM5550388 Pole3KO_H33_CUT_rep1
GSM5550389 Pole4KO_H33_CUT_rep1
GSM5550390 WT_H33_CUT_rep2
GSM5550391 Mcm22A_H33_CUT_rep2
GSM5550392 Pole3KO_H33_CUT_rep2
GSM5550393 Pole4KO_H33_CUT_rep2
GSM5550394 WT_H33_eSPAN_rep1
GSM5550395 Mcm22A_H33_eSPAN_rep1
GSM5550396 Pole3KO_H33_eSPAN_rep1
GSM5550397 Pole4KO_H33_eSPAN_rep1
GSM5550398 WT_H33_eSPAN_rep2
GSM5550399 Mcm22A_H33_eSPAN_rep2
GSM5550400 Pole3KO_H33_eSPAN_rep2
GSM5550401 Pole4KO_H33_eSPAN_rep2
GSM5550402 WT_newTN5_BrdU_rep1
GSM5550403 Mcm22A_newTN5_BrdU_rep1
GSM5550404 Pole3KO_newTN5_BrdU_rep1
GSM5550405 Pole4KO_newTN5_BrdU_rep1
GSM5550406 WT_newTN5_BrdU_rep2
GSM5550407 Mcm22A_newTN5_BrdU_rep2
GSM5550408 Pole3KO_newTN5_BrdU_rep2
GSM5550409 Pole4KO_newTN5_BrdU_rep2

GSM5550410	WT_H3K36me3_CUT_rep1
GSM5550411	Mcm22A_H3K36me3_CUT_rep1
GSM5550412	Pole4KO_H3K36me3_CUT_rep1
GSM5550413	WT_H3K36me3_CUT_rep2
GSM5550414	WT_H3K36me3_eSPAN_rep1
GSM5550415	Mcm22A_H3K36me3_eSPAN_rep1
GSM5550416	Pole4KO_H3K36me3_eSPAN_rep1
GSM5550417	WT_H3K36me3_eSPAN_rep2
GSM5550418	Mcm22A_H3K36me3_eSPAN_rep2
GSM5550419	Pole4KO_H3K36me3_eSPAN_rep2
GSM5550420	WT_BrdU_rep1
GSM5550421	Mcm22A_BrdU_rep1
GSM5550422	Pole4KO_BrdU_rep1
GSM5550423	Pole3KO_RNAseq_rep1
GSM5550424	Pole3KO_RNAseq_rep2
GSM5550425	Pole4KO_RNAseq_rep1
GSM5550426	Pole4KO_RNAseq_rep2
GSM5905548	POLA12A_H33_CUT_rep1
GSM5905549	MCM22APOLE4KO_H33_CUT_rep1
GSM5905550	POLA12A_H33_CUT_rep2
GSM5905551	MCM22APOLE4KO_H33_CUT_rep2
GSM5905552	POLA12A_H33_eSPAN_rep1
GSM5905553	MCM22APOLE4KO_H33_eSPAN_rep1
GSM5905554	POLA12A_H33_eSPAN_rep2
GSM5905555	MCM22APOLE4KO_H33_eSPAN_rep2
GSM5905556	POLA12A_BrdU_rep1
GSM5905557	MCM22APOLE4KO_BrdU_rep1
GSM5905558	POLA12A_BrdU_rep2
GSM5905559	MCM22APOLE4KO_BrdU_rep2
GSM5905560	POLA12A_H3K36me3_eSPAN_rep1
GSM5905561	MCM22APOLE4KO_H3K36me3_eSPAN_rep1
GSM5905562	WT_T0_H33_CR_rep1
GSM5905563	WT_T5_H33_CR_rep1
GSM5905564	WT_T11_H33_CR_rep1
GSM5905565	WT_T0_H33_CR_rep2
GSM5905566	WT_T5_H33_CR_rep2
GSM5905567	WT_T11_H33_CR_rep2

Genome browser session
(e.g. UCSC)

no longer applicable

Methodology

Replicates

Each CUT&Tag, CUT&RUN, eSPAN or RNA-seq was performed in duplicate and showed good agreement.

Sequencing depth

GSM5550386	WT_H33_CUT_rep1	Total reads: 7490287, Unique reads: 4609086, 75bp, pair-end
GSM5550387	Mcm22A_H33_CUT_rep1	Total reads: 7102027, Unique reads: 4467956, 75bp, pair-end
GSM5550388	Pole3KO_H33_CUT_rep1	Total reads: 6593152, Unique reads: 4337553, 75bp, pair-end
GSM5550389	Pole4KO_H33_CUT_rep1	Total reads: 6641846, Unique reads: 4180956, 75bp, pair-end
GSM5550390	WT_H33_CUT_rep2	Total reads: 4688775, Unique reads: 1997356, 75bp, pair-end
GSM5550391	Mcm22A_H33_CUT_rep2	Total reads: 6241940, Unique reads: 3538651, 75bp, pair-end
GSM5550392	Pole3KO_H33_CUT_rep2	Total reads: 3482879, Unique reads: 1875889, 75bp, pair-end
GSM5550393	Pole4KO_H33_CUT_rep2	Total reads: 3854893, Unique reads: 2000388, 75bp, pair-end
GSM5550394	WT_H33_eSPAN_rep1	Total reads: 18051206, Unique reads: 3735599, 75bp, pair-end
GSM5550395	Mcm22A_H33_eSPAN_rep1	Total reads: 22618969, Unique reads: 3859522, 75bp, pair-end
GSM5550396	Pole3KO_H33_eSPAN_rep1	Total reads: 18383107, Unique reads: 4719265, 75bp, pair-end
GSM5550397	Pole4KO_H33_eSPAN_rep1	Total reads: 9622462, Unique reads: 3432837, 75bp, pair-end
GSM5550398	WT_H33_eSPAN_rep2	Total reads: 17847093, Unique reads: 4229620, 75bp, pair-end
GSM5550399	Mcm22A_H33_eSPAN_rep2	Total reads: 21116480, Unique reads: 4129330, 75bp, pair-end
GSM5550400	Pole3KO_H33_eSPAN_rep2	Total reads: 15670799, Unique reads: 4060320, 75bp, pair-end
GSM5550401	Pole4KO_H33_eSPAN_rep2	Total reads: 14844229, Unique reads: 3306575, 75bp, pair-end
GSM5550402	WT_newTN5_BrdU_rep1	Total reads: 8651856, Unique reads: 5248032, 75bp, pair-end
GSM5550403	Mcm22A_newTN5_BrdU_rep1	Total reads: 7202022, Unique reads: 4520389, 75bp, pair-end
GSM5550404	Pole3KO_newTN5_BrdU_rep1	Total reads: 10130608, Unique reads: 5372131, 75bp, pair-end
GSM5550405	Pole4KO_newTN5_BrdU_rep1	Total reads: 13020918, Unique reads: 7663596, 75bp, pair-end
GSM5550406	WT_newTN5_BrdU_rep2	Total reads: 8388243, Unique reads: 5915858, 75bp, pair-end
GSM5550407	Mcm22A_newTN5_BrdU_rep2	Total reads: 9742242, Unique reads: 6931017, 75bp, pair-end
GSM5550408	Pole3KO_newTN5_BrdU_rep2	Total reads: 9363206, Unique reads: 6637605, 75bp, pair-end

GSM5550409 Pole4KO_newTN5_BrdU_rep2 Total reads: 7134556, Unique reads: 4665041, 75bp, pair-end
GSM5550410 WT_H3K36me3_CUT_rep1 Total reads: 11278892, Unique reads: 5718590, 75bp, pair-end
GSM5550411 Mcm22A_H3K36me3_CUT_rep1 Total reads: 10580588, Unique reads: 5294346, 75bp, pair-end
GSM5550412 Pole4KO_H3K36me3_CUT_rep1 Total reads: 8130423, Unique reads: 4141556, 75bp, pair-end
GSM5550413 WT_H3K36me3_CUT_rep2 Total reads: 7636474, Unique reads: 5180256, 75bp, pair-end
GSM5550414 WT_H3K36me3_eSPAN_rep1 Total reads: 23202829, Unique reads: 3667977, 75bp, pair-end
GSM5550415 Mcm22A_H3K36me3_eSPAN_rep1 Total reads: 28834810, Unique reads: 6877429, 75bp, pair-end
GSM5550416 Pole4KO_H3K36me3_eSPAN_rep1 Total reads: 26900265, Unique reads: 5486890, 75bp, pair-end
GSM5550417 WT_H3K36me3_eSPAN_rep2 Total reads: 15362917, Unique reads: 4467284, 75bp, pair-end
GSM5550418 Mcm22A_H3K36me3_eSPAN_rep2 Total reads: 24624939, Unique reads: 5491164, 75bp, pair-end
GSM5550419 Pole4KO_H3K36me3_eSPAN_rep2 Total reads: 30866083, Unique reads: 1713903, 75bp, pair-end
GSM5550420 WT_BrdU_rep1 Total reads: 22319455, Unique reads: 5203394, 75bp, pair-end
GSM5550421 Mcm22A_BrdU_rep1 Total reads: 21282712, Unique reads: 4576832, 75bp, pair-end
GSM5550422 Pole4KO_BrdU_rep1 Total reads: 13043595, Unique reads: 4012653, 75bp, pair-end
GSM5550423 Pole3KO_RNAseq_rep1 Total reads: 37357395, Unique reads: 30804424, 200bp, pair-end
GSM5550424 Pole3KO_RNAseq_rep2 Total reads: 39152583, Unique reads: 32854901, 200bp, pair-end
GSM5550425 Pole4KO_RNAseq_rep1 Total reads: 55152336, Unique reads: 45428036, 200bp, pair-end
GSM5550426 Pole4KO_RNAseq_rep2 Total reads: 49353135, Unique reads: 40576961, 200bp, pair-end
GSM5905548 POLA12A_H33_CUT_rep1 Total reads: 6887146, Unique reads: 3942605, 75bp, pair-end
GSM5905549 MCM22APOLE4KO_H33_CUT_rep1 Total reads: 7100002, Unique reads: 4041400, 75bp, pair-end
GSM5905550 POLA12A_H33_CUT_rep2 Total reads: 8591351, Unique reads: 4826993, 75bp, pair-end
GSM5905551 MCM22APOLE4KO_H33_CUT_rep2 Total reads: 7302055, Unique reads: 3864367, 75bp, pair-end
GSM5905552 POLA12A_H33_eSPAN_rep1 Total reads: 18735554, Unique reads: 5067027, 75bp, pair-end
GSM5905553 MCM22APOLE4KO_H33_eSPAN_rep1 Total reads: 26639273, Unique reads: 8140456, 75bp, pair-end
GSM5905554 POLA12A_H33_eSPAN_rep2 Total reads: 7419005, Unique reads: 3823637, 75bp, pair-end
GSM5905555 MCM22APOLE4KO_H33_eSPAN_rep2 Total reads: 9788830, Unique reads: 1993823, 75bp, pair-end
GSM5905556 POLA12A_BrdU_rep1 Total reads: 18091199, Unique reads: 12995032, 75bp, pair-end
GSM5905557 MCM22APOLE4KO_BrdU_rep1 Total reads: 14117737, Unique reads: 10007173, 75bp, pair-end
GSM5905558 POLA12A_BrdU_rep2 Total reads: 7386117, Unique reads: 5357626, 75bp, pair-end
GSM5905559 MCM22APOLE4KO_BrdU_rep2 Total reads: 6262127, Unique reads: 4490492, 75bp, pair-end
GSM5905560 POLA12A_H3K36me3_eSPAN_rep1 Total reads: 39482543, Unique reads: 5551911, 75bp, pair-end
GSM5905561 MCM22APOLE4KO_H3K36me3_eSPAN_rep1 Total reads: 33815374, Unique reads: 6515747, 75bp, pair-end
GSM5905562 WT_T0_H33_CR_rep1 Total reads: 17700119, Unique reads: 12486731, 75bp, pair-end
GSM5905563 WT_T5_H33_CR_rep1 Total reads: 4989870, Unique reads: 3208015, 75bp, pair-end
GSM5905564 WT_T11_H33_CR_rep1 Total reads: 8170789, Unique reads: 5113763, 75bp, pair-end
GSM5905565 WT_T0_H33_CR_rep2 Total reads: 14854800, Unique reads: 8922495, 75bp, pair-end
GSM5905566 WT_T5_H33_CR_rep2 Total reads: 14573217, Unique reads: 8850387, 75bp, pair-end
GSM5905567 WT_T11_H33_CR_rep2 Total reads: 13170379, Unique reads: 7253758, 75bp, pair-end

Antibodies

Antibodies used for CUT&Tag, eSPAN and CUT&RUN in this study including: anti-Biotin (A150-109A, Bethyl), anti-H3K36me3 (61021, Active Motif) and anti-BrdU (555627, BD Biosciences).

Peak calling parameters

CUT&Tag and CUT&RUN reads aligned to mouse (mm10) reference genome using Bowtie2 (version 2.2.4) with --no-mixed --no-discordant --no-dovetail --no-contain --local parameters. Islands were called using SICER2 (version 1.0.3) tool with -s mm10 -w 5000 -f 200 -fdr 0.001 -g 10000 -cpu 50 -rt 0 parameters for CUT&RUN data

Data quality

Sequencing adaptors and Low-quality reads (q < 20) were removed using Trim Galore. CUT&Tag, CUT&RUN and RNA-seq experiment were performed with two repeats.

Software

CUT&Tag reads were aligned to mm10 using Bowtie2 allowing up to 1 mismatches, discarding ambiguous and clonal reads. CUT&RUN reads were aligned to mm10 and hg19 reference separately using Bowtie2. RNA-seq reads were aligned to the mouse genome (GENCODE mm10 primary assembly) using STAR (version 2.7.6a).

Flow Cytometry

Plots

Confirm that:

- [x] The axis labels state the marker and fluorochrome used (e.g. CD4-FITC).
- [x] The axis scales are clearly visible. Include numbers along axes only for bottom left plot of group (a 'group' is an analysis of identical markers).
- [x] All plots are contour plots with outliers or pseudocolor plots.
- [x] A numerical value for number of cells or percentage (with statistics) is provided.

Methodology

Sample preparation	For cell cycle analysis, exponentially growing mouse E14 cells were collected and fixed in 70% ethanol in PBS, then rotated at 4°Covernight. Cells were washed once with PBS and incubated in PI staining solution. For Annexin V stain, cells were collected and resuspended in Annexin V binding buffer, incubated with Annexin V conjugate and propidium iodide for 15mins according to protocols and then subjected to Attune flow cytometer.
Instrument	LSR II flow cytometer (BD Biosciences). Attune (ThermoFisher)
Software	BD FACSDiva and Attune NxT Cytometric softwares were used for collecting data. Data were analyzed by Flowjo (version 10.7.1) or FCS Express 7.
Cell population abundance	10,000 cells from each sample were used for cell cycle analysis. We used FSC vs SSC gating method to exclude cell debris. Live cell purity were between 60-90%.
Gating strategy	Forward versus side scatter (FSC vs SSC) gating is used to identify cells of interest based on size and granularity. Basically, we gate upper right population to exclude debris as cell debris tend to be found at the bottom left corner of the FSC vs SSC density plot. A forward scatter height (FSC-H) vs. forward scatter area (FSC-A) density plot is used to exclude doublets in cell cycle assay. A forward light scatter width (FSC-W) vs. forward scatter area (FSC-A) density plot is used to exclude doublets in Annexin V apoptosis detection assay. For detection of apoptosis and necrotic cells, Annexin V signal was compared to PI signal (cell permeability). To separate PI positive and Annexin V positive cells, samples without PI or Annexin V were measured as negative controls.

☒ Tick this box to confirm that a figure exemplifying the gating strategy is provided in the Supplementary Information.

Magnetic resonance imaging

Experimental design

Design type	*Indicate task or resting state; event-related or block design.*
Design specifications	*Specify the number of blocks, trials or experimental units per session and/or subject, and specify the length of each trial or block (if trials are blocked) and interval between trials.*
Behavioral performance measures	*State number and/or type of variables recorded (e.g. correct button press, response time) and what statistics were used to establish that the subjects were performing the task as expected (e.g. mean, range, and/or standard deviation across subjects).*

Acquisition

Imaging type(s)	*Specify: functional, structural, diffusion, perfusion.*
Field strength	*Specify in Tesla*
Sequence & imaging parameters	*Specify the pulse sequence type (gradient echo, spin echo, etc.), imaging type (EPI, spiral, etc.), field of view, matrix size, slice thickness, orientation and TE/TR/flip angle.*
Area of acquisition	*State whether a whole brain scan was used OR define the area of acquisition, describing how the region was determined.*

Diffusion MRI ☐ Used ☐ Not used

Preprocessing

Preprocessing software	*Provide detail on software version and revision number and on specific parameters (model/functions, brain extraction, segmentation, smoothing kernel size, etc.).*
Normalization	*If data were normalized/standardized, describe the approach(es): specify linear or non-linear and define image types used for transformation OR indicate that data were not normalized and explain rationale for lack of normalization.*
Normalization template	*Describe the template used for normalization/transformation, specifying subject space or group standardized space (e.g. original Talairach, MNI305, ICBM152) OR indicate that the data were not normalized.*
Noise and artifact removal	*Describe your procedure(s) for artifact and structured noise removal, specifying motion parameters, tissue signals and physiological signals (heart rate, respiration).*
Volume censoring	*Define your software and/or method and criteria for volume censoring, and state the extent of such censoring.*

Statistical modeling & inference

Model type and settings	*Specify type (mass univariate, multivariate, RSA, predictive, etc.) and describe essential details of the model at the first and second levels (e.g. fixed, random or mixed effects; drift or auto-correlation).*

Effect(s) tested

Define precise effect in terms of the task or stimulus conditions instead of psychological concepts and indicate whether ANOVA or factorial designs were used.

Specify type of analysis: ☐ Whole brain ☐ ROI-based ☐ Both

Statistic type for inference
(See Eklund et al. 2016)

Specify voxel-wise or cluster-wise and report all relevant parameters for cluster-wise methods.

Correction

Describe the type of correction and how it is obtained for multiple comparisons (e.g. FWE, FDR, permutation or Monte Carlo).

Models & analysis

n/a	Involved in the study
☐	☐ Functional and/or effective connectivity
☐	☐ Graph analysis
☐	☐ Multivariate modeling or predictive analysis

Functional and/or effective connectivity

Report the measures of dependence used and the model details (e.g. Pearson correlation, partial correlation, mutual information).

Graph analysis

Report the dependent variable and connectivity measure, specifying weighted graph or binarized graph, subject- or group-level, and the global and/or node summaries used (e.g. clustering coefficient, efficiency, etc.).

Multivariate modeling and predictive analysis

Specify independent variables, features extraction and dimension reduction, model, training and evaluation metrics.
